# Editorial: Music and Cochlear Implants: Recent Developments and Continued Challenges

**DOI:** 10.3389/fnins.2021.736772

**Published:** 2021-08-12

**Authors:** Alexandre Lehmann, Charles J. Limb, Jeremy Marozeau

**Affiliations:** ^1^BRAMS-CRBLM, McGill University Faculty of Medicine, Montreal, QC, Canada; ^2^School of Medicine, University of California, San Francisco, San Francisco, CA, United States; ^3^Technical University of Denmark, Kongens Lyngby, Denmark

**Keywords:** music, cochlear implants, cognition, sensory deprivation, hearing restoration

Cochlear implants (CI) rank as the most successful neural prostheses. They can restore hearing in severe-to-profound hearing-impaired individuals, whether congenitally or post-lingually deafened. Almost a million patients worldwide have received a cochlear implant. Unlike traditional hearing aids, cochlear implants do not amplify sounds. They electrically stimulate the auditory nerve directly, thereby sending signals to the brain that can be perceived as sounds. Although most CI patients achieve some level of speech perception, many experience very poor music perception, both in terms of self-reported music enjoyment and objective perceptual abilities, which are significantly lower than in normal hearing subjects (Marozeau et al., 2014).

Far from being “auditory cheesecake,” music is an important part of social life, well-being, and quality of life. From prehistory to the present, and across all known cultures, music has always played an important role in social gatherings and mood regulation (Huron, 2008). Listening to music with friends, singing in a religious event, playing an instrument, or attending live music events are things with which many cochlear implant patients struggle. Recent evidences also point to music as an important medium for developing the human brain—both in terms of cognitive, emotional, and auditory-motor processing abilities (Thaut and Hodges, 2021).

However, decades of research and development on signal processing, stimulation, perception, and rehabilitation in cochlear implant recipients have focused mainly on speech. Substantial research is needed to give cochlear implant recipients better access to music and its numerous benefits. To stimulate and synthesize this expanding field of research, a group of researchers from all over the world gathered at McGill University in Montreal, Canada in August 2018 for the second international symposium on Music and Cochlear Implants. During two stimulating days, attendees presented and discussed recent developments and current challenges in music perception, appreciation, and music-based rehabilitation. A unique aspect of the symposium was that six cochlear implant recipients, trained at a high level of musicianship, answered our call for participation. They shared very moving testimonials and were featured on a dedicated panel discussing their experiences with music. Their stories made an everlasting impression on all the attendants. A unique aspect of this Research Topic is the involvement and co-authoring of these six musicians CI users in a patient-centered article by music and implant pioneer Dr. Gfeller from the University of Iowa.

Several attendees at this meeting contributed to this Research Topic; other research groups have added contributions sharing related ideas. The present Research Topic thus provides an excellent overview of the current state of the art in music and cochlear implants. The music cognition literature makes an important distinction between music perception and music appraisal, the former being about the objectively measured capacity to process certain sound features. In contrast, the latter is about the listener's subjective experience of music (Looi et al., 2012). This Research Topic covers the two main dimensions of music perception and music appraisal, in such diverse fields as psychoacoustics, psycholinguistics, electrophysiology, audiology, signal processing, music psychology as well as qualitative and patient-engaged research, both in pediatric and adult CI recipients.

Starting with the perceptual aspect of music, Erickson et al. used multidimensional scaling to investigate timbre perception in normal-hearing participants listening to vocoded stimuli to simulate CI hearing. Also, using a vocoded approach, Luo and Hayes asked whether supplementing hearing with vibrotactile stimulation can improve melody identification. All other studies in this Research Topic were conducted with cochlear implant recipients, in some cases matched with normal-hearing controls. Zimmer et al. examined musical harmony and syntax in pediatric pre-lingually deaf CI users, using psychoacoustic discrimination and preference of typical musical chords. Also using musical chords, but in a linguistic priming task, Tillmann et al. investigated implicit processing of pitch in post-lingually deafened CI adults. Using a continuous rating approach, Spangmose et al. evaluated CI users' ability to perceive musical tension, an important high-level feature in appreciating music. Looking at important low-level features, Swanson et al. compared pitch and melody perception using place of excitation and temporal cues. One of the challenges in this field is using appropriate tools to assess music perception in the CI population, Steel et al. created a modified version of a popular music cognition battery by manipulating the music excerpt's timbre and spectrum to account for the technological limitation of cochlear implants. To complement behavioral measures with objective physiological recordings, Petersen et al. have introduced a new paradigm using the mismatch negativity response to test music discrimination in CI users. Given the importance of bass frequencies in music cognition, several studies looked at the impact of additional low-frequency access in patients. Yüksel et al. assessed the effect of low-frequency residual hearing on pediatric CI users' music perception. D'Onofrio et al. examined the impact of combining electric (cochlear implant) and contralateral acoustic (hearing aid) stimulation on the perception of musical emotion in so-called “bimodal” patients. Two studies took advantage of the unique research opportunities offered by so called “single-sided-deafness” patients, who have normal hearing on one ear and a cochlear implant in the contralateral ear and can therefore perform direct perceptual comparisons between normal-hearing and cochlear-implant hearing. Spitzer et al. compared dissonance ratings of harmonic interval between the normal hearing and CI ears. Adel et al. examined the effects of electrode position and acoustic stimulus type on a classical pitch-matching task used in this population.

Assessing the subjective music experience, as well as perceptual abilities, Fuller et al. addressed a long-standing question of music appraisal differences between pre- and post-lingually deafened individuals and how it relates to perceptual skills. The next four studies focus on the experience and appraisal of music. Berg et al. measured the perceived sound quality and its relation to the number of implant channels. An approach to improve music appraisal in CI users is to modify the actual music signal. Gauer et al. examined how a music-pre-processing scheme based on spectral complexity reduction impacted music enjoyment in CI users. Tahmasebi et al. designed a real-time music processing algorithm and examined the impact of independently adjust the loudness of the vocals in the music on CI users' enjoyment. The last three studies of this Research Topic went beyond the lab, focusing on every day musical experiences of CI users and their family, using questionnaires, interviews and patient–engaged methodology. Gfeller, Driscoll, et al. explored the perspectives of adult CI recipients regarding two experiences with music in everyday life: music listening and background music that competes with spoken conversation. Looi et al. examined the role and importance placed on music by families with normally hearing children compared to hearing-impaired children. Last but not least, another contribution by Gfeller, MacMullen Mallalieu et al. involved a unique collaboration and co-authorship of six CI users engaged in high levels of musicianship who participated in the Music & CI symposium. It is also the only contribution from this Research Topic that looks at music-making. It documents personal characteristics and experiences and suggests possible strategies useful to other CI users interested in improving music experiences.

Taken together, the contributions in this Research Topic are a first step in driving this new exciting field in the making, and we hope it may inspire new research that addresses many of the pending fundamental and clinical questions on the topic. For instance, why do some CI users have “supernatural” pitch discrimination abilities given the current technical and biological constraints (Maarefvand et al., 2013; Limb and Roy, 2014)? Should clinicians try to improve music perception or rather focus on eliciting an equivalent emotional response to music (Paquette et al., 2018)? How can we bring research toward more ecological, real-life-like situations to understand the patient experience better? Finally, how does one advocate dedicating time for music when resources are already severely limited for speech-focused interventions?

## Author Contributions

All authors contributed to manuscript writing and approved the submitted version.

## Conflict of Interest

CL has been acting as a consultant for and has received grant support from Advanced Bionics, MED-EL and Oticon Medical. JM has received grant support from Oticon Medical. AL has received grant support from MED-EL and Oticon Medical.

## Publisher's Note

All claims expressed in this article are solely those of the authors and do not necessarily represent those of their affiliated organizations, or those of the publisher, the editors and the reviewers. Any product that may be evaluated in this article, or claim that may be made by its manufacturer, is not guaranteed or endorsed by the publisher.
